# Variations in basic demographics consequential to population size of governorate in Saudi Arabia

**DOI:** 10.1186/s40064-016-3126-0

**Published:** 2016-08-30

**Authors:** Rshood Khraif, Asharaf Abdul Salam, Rajaram Subramanian Potty, Ali Aldosari, Ibrahim Elsegaey, Abdullah AlMutairi

**Affiliations:** 1 Center for Population Studies, King Saud University, Riyadh, Saudi Arabia; 2 Karnataka Health Promotion Trust, Bangalore, India

**Keywords:** Coefficient of variation, Administrative units, Population ratios, Immigration, Native versus foreign population

## Abstract

Saudi Arabia, divided into 5 planning regions, 13 administrative regions and further to 118 governorates (administrative units), has diverse demographic characteristics from one region to another and from one governorate to another. Rural to urban migration and an exodus of immigrants characterize the Kingdom, where development planning depend largely upon local level requirements based on economic activities. An attempt was made to analyze the population characteristics, such as population size, sex ratio, native to foreigner ratio, and households and persons per households by keeping governorate as unit of analysis. Data of two census period (2004 and 2010) was used in order to explore the situation and track the intercensal changes. Large variations in population were observed between governorates and it varied from 3686 to 5,007,886 in 2010. Governorates are divided according to the number of native population demarcating urbanization, modernization and infrastructure. During the intercensal period, the number of small governorates reduced and medium and large sized governorates increased mainly due to population growth. The average population in governorates was increased in total and in the larger governorates during the period. However, we noticed a reduction in the average population size in the small and medium sized governorates. The size of native population in a governorate influences the sex ratio, the native-foreigner ratio and the persons per household as well as the variations within the group of governorates. Analyses of lower level data shall aid not only to understand the situation but also to support local development policies.

## Background

There has been a change in all dimensions of demography including population growth, immigration, urbanization, age distribution and labor force participation in Saudi Arabia, which is the largest country in the Arabian Peninsula (Alrouh et al. 2013). These changes lead to modifications in the socio-economic conditions and infrastructure—housing, education and health. The population growth is the result of the natural increase, traditional Islamic culture (Wincker 1997) and labor oriented immigrations (Khraif 2000, 2007) along the improvement in living conditions, quality of life and infrastructure resulting from economic prospects and urbanization (Susilawati and Al-Surf 2011).

Demographically, Saudi Arabia encourages pronatalist policies that create high fertility despite the prevailing low mortality (Freedman 1995; Jacobson 1994; Omran and Roudi-Fahimi 1993). However, birth rates declined as a result of educational improvements and increasing age at marriage in the Kingdom (Al-Mazrou et al. 1995; Al-Nasser and Bamgboye 1992). The Kingdom has 7.4 % of 359 million Arabs—65.1 % of the Gulf Cooperation Council-GCC (Alrouh et al. 2013; Center for Population Studies 2013; Rashad 2000). The undergoing fertility transition (Alrouh et al. 2013; Khraif 2009; Courbage 1999) of the Kingdom can be attributed to the improvement in health status (United Nations 2002; Shawky 2001). But the existing youth bulge and immigration of foreign laborers (Collemore 2003; Samman 1985); unbalanced sex ratio (Parasuraman 2002); regional disparities in physical and social infrastructure (Al-Khalifeh 1993); increased demands for education, housing, health care and employment (Roudi-Fahimi 1993); and residential mobility (Khraif 1994) in the rapidly urbanizing Saudi Arabia (Susilawati and Al-Surf 2011; Sly and Serow 1993) receive attention of both reformers and policy makers. More than three-fifths of the population of Saudi Arabia lives in major cities, where networks of transportation as well as most basic services are well organized and integrated.

The alarmingly urbanizing Kingdom of Saudi Arabia (Khraif 2007) has relatively integrated transportation networks and most basic services (UNDP 2004) addressing the increasing demand for public services such as piped water, electricity, sewage, telephone (United Nations 2006; Makki 1986), housing and transportation (Al-Gabbani 2008) even though with wide spread disparities in physical and social infrastructure (Al-Khalifeh 1993) observed across administrative areas. Local level variations visibly based upon not only the geographic characteristics but also the developmental paradigms are noted. Saudi Arabia has an East West Corridor of development (Khraif 2007) connecting Eastern Region, Al-Riyadh, Al-Madina Al-Monawarah and Makkah Al-Mokarramah, having residential, commercial, and industrial and port networks. This population concentrated corridor has the Kingdom’s majority of developmental activities—education, health, road, and housing infrastructure—demanding both national and foreign labor force. Other regions, on the north and the south, having low population density comprising of lesser commercial and industrial activities; thus having less demand for labor force.

Within the regions, governorates differ widely in terms of population size including national and expatriate, and infrastructure—education, health and other utilities. It is the population size in a given region that determines other factors reflecting demographic, social, economic, and health conditions. Demographic indicators such as sex ratio, native-expatriate ratio and persons per household has significance not only in the changing migration and labor laws in the Kingdom but also in the regional development and population redistribution efforts. It is in this context, an analysis of governorates of the Kingdom of Saudi Arabia is carried out using 2004 and 2010 census data.

## Data and methodology

The present paper provides comparable and most recent data on changes on the current characteristics of the population in Saudi Arabia by regions and groups of governorates according to their population size. The census data (published by the Ministry of Economics and Planning) for the years 2004 and 2010 was analyzed in detail using MSExcel and SPSS (version 20), and applied suitable statistical methods. Governorates were classified into three such as small (<50,000 population), medium (50,000–100,000) and large (100,000+), according to the size of Saudi population, have been analyzed keeping governorate as the unit of quantifying Saudi and non-Saudi population; sex ratio; Saudi-non-Saudi ratio; and average number of persons per household. In addition, we also calculated the coefficient of variation to find out the difference in the population parameters between various governorates as per the classification of Saudi population. Caution is required while interpreting the totals in comparison with the categories based on Saudi population, across the census years, as the denominator (number of governorates) differs, due to the use of preliminary and final census (2010).

## Results and discussions

Data analyses have been made in this paper to explore population size, sex ratio, native-foreigner ratio, number of households and persons per household.

### Population of governorates

Governorates, the administrative units, in Saudi Arabia have not been formed according to the population size but to the local level development requirements. In total, there are 118 governorates from the thirteen regions of the country (see Table 1). The number of governorates are highest in the Al-Riyadh region (20) followed by Jazan (14), Makkah Al-Mokarramah (12), Aseer (12), Al-Qaseem (11) and Eastern Region (11). The number of governorates is least in Al-Jouf (3), Northern Borders (3) and Hail (4). According to the census 2004, 62 governorates (52.5 %) in Saudi Arabia, have less than 50,000 native population; 24 governorates (20.4 %) have native population of size between 50,000 and 100,000 and the remaining 32 governorates (27.1 %) have 100,000 or more native population. The number of governorates with the same native population size was changed to 50 (42.4 %), 32 (27.1 %) and 36 (30.5 %) in the year 2010. Thus there is a reduction of 10 percentage point in the number of small governorates between the two census periods. Consequently, the number of medium and large governorates increased by about 7 and 3 percentage points, respectively. These governorates have both urban and rural areas with increasing inhabitation in small towns and medium sized cities having investments in urban development (United Nations 2006; UNDP 2004; Sly and Serow 1993) leading to huge expansion and rapid growth of cities in the Kingdom (Khraif 1994). In the year 2004, no governorate was identified as medium size in the Eastern Region, Tabouk and Al-Jouf. However, in the year 2010, the regions of Al-Qaseem and Al-Jouf did not have any medium sized governorate. Similarly, none of the governorates in Al-Baha region had native population of 100,000 or more in the year 2004 and 2010.

**Table 1:** Distribution of governorates by Saudi population size, across regions

Region	2004			2010			Total
	<50,000	50,000–100,000	100,000+	<50,000	50,000–100,000	100,000+	
Al-Riyadh	11 (55.0)	6 (30.0)	3 (15.0)	11 (55.0)	4 (20.0)	5 (25.0)	20 (100.0)
Makkah Al-Mokarramah	6 (50.0)	1 (8.3)	5 (41.7)	5 (41.7)	2 (16.7)	5 (41.7)	12 (100.0)
Al-Madina Al-Monawarah	4 (57.1)	1 (14.3)	2 (28.6)	1 (14.3)	4 (57.1)	2 (28.6)	7 (100.0)
Al-Qaseem	8 (72.7)	1 (9.1)	2 (18.2)	8 (72.7)	–	3 (27.3)	11 (100.0)
Eastern Region	5 (45.5)	–	6 (54.5)	4 (36.4)	1 (9.1)	6 (54.5)	11 (100.0)
Aseer	3 (25.0)	5 (41.7)	4 (33.3)	1 (8.3)	6 (50.0)	5 (41.7)	12 (100.0)
Tabouk	5 (83.3)	–	1 (16.7)	4 (66.7)	1 (16.7)	1 (16.7)	6 (100.0)
Hail	2 (50.0)	1 (25.0)	1 (25.0)	2 (50.0)	1 (25.0)	1 (25.0)	4 (100.0)
Northern Borders	1 (33.3)	1 (33.3)	1 (33.3)	1 (33.3)	1 (33.3)	1 (33.3)	3 (100.0)
Jazan	8 (57.1)	2 (14.3)	4 (28.6)	3 (21.4)	7 (50.0)	4 (28.6)	14 (100.0)
Najran	6 (75.0)	1 (12.5)	1 (12.5)	6 (75.0)	1 (12.5)	1 (12.5)	8 (100.0)
Al-Baha	3 (42.9)	4 (57.1)	–	3 (42.9)	4 (57.1)	–	7 (100.0)
Al-Jouf	1 (33.3)	–	2 (66.7)	1 (33.3)	–	2 (66.7)	3 (100.0)
Total	62 (52.5)	24 (20.3)	32 (27.1)	50 (42.4)	32 (27.1)	36 (30.5)	118 (100.0)

Thus, the increase in population per governorate reflects the increase in population during the intercensal period, due mainly to the natural increases. Governorates grouped according to the number of native population, shows an increasing trend of governorates by population. This, on the other hand indicates local level developments, urban growth, and expansion of small and medium sized governorates, during the intercensal period 2004–2010.

In the year 2010 (Final Results), total population of 26.1 million persons lived in 4,652,162 housing units in Saudi Arabia. Out of the total population, 19.3 million are native persons (74.1 %) lived in 2,996,253 housing units (the preliminary results shows a total population of 27.1 million, out of which 18.7 million are Saudi—68.9 %—living in 4.6 million housing units). Comparatively, as per the census 2004, the total population was about 22.7 million, lived in 3,991,783 housing units. The count of native population was about 16.5 million (72.9 %) lived in 2,761,738 housing units. The remaining 27.1 % in the year 2004 and 25.9 % in the year 2010 were foreigners, mostly from South Asian and African countries.

It shows an alarming increase of expatriate population in the Kingdom during the intercensal period of 2004–2010. Consequently, the significant proportion of expatriate population, characteristic of GCC nations, exerts pressure on social and economic life leading to unemployment of natives as well as shortage of basic supplies (Khraif 2009; Collemore 2003). Such an influx of foreign labor has developed from the scenario of a small number of national population employed in public sector that attracts foreign labor in other sectors of infrastructure development—power stations, government ministries and services, and industrial and agricultural units (Wincker 1997), though the situation undergoing rapid changes recently. The population in Saudi Arabia was grown by 2.5 persons annually during the period 2004–2010. However, during the same period the native population was grown by 2.8 persons annually. Thus, establishes the population growth from 2004 to 2010 affecting housing units, native to foreigner population, and the labor force composition; consequently, influencing the national demographic scenario.

### Population size

We calculated the average population size in the governorates and the results are provided in the Table 2. Overall, in the year 2010, the governorates in the Kingdom had a population size of 221,106 persons per governorate, on an average (163,859 native and 57,247 non-native), and the average population size in 2004 was 192,189 persons per governorates (140,062 native and 52,126 non-native). While the small governorates had a mean population of 35,839 and 34,196; medium governorates had 76,818 and 74,265 and large governorates had 581,645 and 611,226 in the year 2004 and 2010, respectively. The result thus indicates that the population growth during the inter-census period was mainly at the large governorates. In each class of governorates, the population size of non-native persons is proportional to population size of native persons. For example, in both study years large governorates had higher number of non-native persons. In other words, a higher number of native persons in a governorate boost the size of non-native persons.

**Table 2:** Average population of governorate, across regions

Region	Saudi	Non Saudi	Total	Saudi	Non Saudi	Total	Saudi	Non Saudi	Total	Saudi	Non Saudi	Total
	<50,000			50,000–100,000			100,000+			Total		
a. Average population size												
*2004*												
Al-Riyadh	20,035	7520	27,555	68,658	15,143	83,801	1,031,075	519,711	1,550,786	186,278	86,636	272,914
Makkah Al-Mokarramah	33,756	5018	38,774	58,728	13,555	72,282	659,679	432,071	1,091,750	298,719	184,380	483,099
Al-Madina Al-Monawarah	46,990	5315	52,305	51,885	6203	58,088	452,213	170,495	622,708	163,467	52,636	216,103
Al-Qaseem	26,360	5667	32,027	94,361	21,520	115,881	256,015	65,924	321,938	74,297	18,064	92,361
Eastern Region	33,730	8946	42,676	–	–	–	397,809	126,633	524,442	232,318	73,139	305,457
Aseer	44,057	6393	50,451	66,306	8484	74,790	242,785	47,874	290,659	119,570	21,091	140,662
Tabouk	35,274	5396	40,670	–	–	–	417,901	70,464	488,365	99,045	16,241	115,286
Hail	31,961	5830	37,791	44,474	2792	47,266	298,879	57,891	356,770	112,937	18,784	131,721
Northern Borders	36,107	5678	41,785	63,407	9956	73,363	140,320	24,503	164,823	79,945	13,379	93,324
Jazan	37,288	6323	43,612	58,277	8163	66,440	144,791	31,663	176,454	71,002	13,826	84,828
Najran	12,330	1317	13,647	62,820	10,145	72,965	212,240	53,258	265,498	43,630	8913	52,543
Al-Baha	30,594	3840	34,434	59,134	9516	68,650	–	–	–	46,902	7083	53,986
Al-Jouf	34,608	5461	40,069	–	–	–	136,713	24,122	160,835	102,678	17,901	120,579
Total	30,063	5776	35,839	65,644	11,174	76,818	409,000	172,645	581,645	140,062	52,126	192,189
*2010*												
Al-Riyadh	22,558	8147	30,704	66,247	13,782	80,029	798,639	370,890	1,169,528	225,316	99,959	325,275
Makkah Al-Mokarramah	36,705	5404	42,109	68,089	17,770	85,860	791,862	464,204	1,256,066	356,585	198,631	555,216
Al-Madina Al-Monawarah	44,074	3953	48,027	55,513	6649	62,162	514,895	184,141	699,036	185,131	56,976	242,107
Al-Qaseem	30,754	6021	36,775	–	–	–	237,129	59,592	296,722	87,038	20,631	107,670
Eastern Region	34,697	8913	43,610	57,537	9512	67,049	462,553	130,494	593,047	270,150	75,284	345,434
Aseer	44,901	8902	53,803	64,234	7522	71,756	239,223	43,356	282,579	135,535	22,568	158,103
Tabouk	34,496	5387	39,882	54,839	6479	61,318	479,880	76,952	556,832	112,117	17,496	129,613
Hail	35,173	6849	42,022	97,358	6090	103,448	339,896	65,919	405,815	126,900	21,427	148,327
Northern Borders	41,135	6720	47,855	70,432	7834	78,266	162,683	22,669	185,352	91,417	12,408	103,824
Jazan	15,792	734	16,527	63,772	10,133	73,906	156,936	34,399	191,336	80,109	15,052	95,162
Najran	13,264	1475	14,739	73,435	11,014	84,449	256,487	67,245	323,732	51,188	10,888	62,077
Al-Baha	33,678	4030	37,708	63,388	10,012	73,400	–	–	–	50,655	7448	58,103
Al-Jouf	40,640	7936	48,576	–	–	–	158,913	30,931	189,845	119,489	23,266	142,755
Total	28,471	5727	34,196	64,443	9822	74,265	440,268	170,959	611,226	163,859	57,247	221,106

This trend—native to expatriate widened during the intercensal period, despite concerted efforts in line with nationalization of labor force. Such changes during the intercensal period are of concern to the demographers and other policy oriented researchers as the increasing trend of expatriates in the Kingdom shall have long term implications on population distribution and family formations.

Overall, both in 2004 and 2010, the average population of the native persons per governorate was highest in Makkah Al-Mokarramah, followed by Al-Riyadh and Eastern Region. However, in 2004 Najran, Al-Baha, and Jazan had lowest mean population of the native persons per governorate. In 2010, lowest average population of native persons per governorate was in Al-Baha followed by Najran and Jazan. As per the 2004 census, the average population size of native persons in the small governorates was lowest in the Najran region followed by Al-Riyadh, Al-Qaseem, Al-Baha and Hail. In case of medium governorates, the average population size of native persons was lowest in the region Al-Madina Al-Monawarah followed by Jazan, Makkah Al-Mokarramah and Al-Baha. On the other hand, Al-Riyadh and Makkah Al-Mokarramah had the highest average native persons in the governorates classified as large. Mass migrations from rural to urban areas increasing over urbanization, alarm the growth of primate city (Makki 1986)—Riyadh, which is the fastest growing city in the Middle East (Susilawati and Al-Surf 2011), where a large number of non-native persons are brought for employment (Center for Population Studies 2013).

Population size in these governorates has increased markedly during the intercensal period, in line with population growth of the Kingdom, in general. It is the medium and large governorates whose population expanded remarkably in the period. It shows the governmental efforts in line with development in both medium and large governorates, neglecting the smaller ones.

### Sex ratio

Sex ratio as provided by the census was examined and it was found that the over all sex ratio was 132 in 2010 but 124 in 2004 (Table 3), showing an increasing trend. It ranged from 118 to 134 in different governorates classified according to their population size of native persons in 2010, whereas it ranged between 112 and 126 in 2004; again an increasing trend. While we identified a balanced sex ratio of 104 males per 100 females among the native persons in 2010 it ranged from 98 to 106 in governorates as per their population size of native persons. However, in 2010 an imbalanced sex ratio of 238 males per 100 females was identified for non-native persons and the highest sex ratio among the non-native persons was in the small governorates. This higher sex ratios of the expatriates is alarming indicating the male dominating labor sector in the Kingdom and its requirements, especially in the smaller governorates. In the medium and larger governorates, labor force demands more technical and professional expatriates accommodating families.

**Table 3:** Sex ratio at governorate, across regions

Region	Saudi	Non Saudi	Total	Saudi	Non Saudi	Total	Saudi	Non Saudi	Total	Saudi	Non Saudi	Total
	<50,000			50,000–100,000			100,000+			Total		
*2004*												
Al-Riyadh	100	448	141	95	401	119	106	227	135	104	239	134
Makkah Al-Mokarramah	89	394	105	96	383	121	101	178	126	100	181	125
Al-Madina Al-Monawarah	91	535	106	96	458	110	99	189	118	98	201	116
Al-Qaseem	97	511	124	96	308	117	101	352	127	99	375	125
Eastern Region	104	392	132	–	–	–	105	291	132	105	295	132
Aseer	86	358	101	91	449	106	97	338	117	102	354	113
Tabouk	98	386	115	–	–	–	109	292	124	106	314	122
Hail	94	687	120	93	635	102	95	288	112	95	338	111
Northern Borders	99	348	115	97	334	113	102	279	117	100	301	115
Jazan	96	203	106	94	200	103	95	199	108	95	200	107
Najran	94	525	108	110	244	122	98	246	116	99	264	115
Al-Baha	83	425	97	89	310	104	–	–	–	87	332	102
Al–Jouf	97	335	113	–	–	–	101	312	118	100	315	117
Total	95	384	116	94	358	112	102	216	126	101	227	124
*2010*												
Al-Riyadh	104	443	153	98	427	126	108	237	143	107	246	143
Makkah Al-Mokarramah	92	380	111	101	588	150	103	184	131	103	188	130
Al-Madina Al-Monawarah	90	587	105	94	479	112	103	201	127	101	213	124
Al-Qaseem	101	491	134	–	–	–	103	319	132	103	349	133
Eastern Region	106	475	156	106	397	144	108	312	144	108	319	144
Aseer	90	308	112	92	416	109	101	320	122	99	333	119
Tabouk	101	409	124	97	384	113	108	292	126	106	317	124
Hail	99	687	136	–	–	–	99	294	118	99	326	121
Northern Borders	101	389	125	95	330	112	103	285	121	101	309	119
Jazan	169	247	173	101	222	114	100	208	116	103	213	117
Najran	98	538	117	108	280	123	101	247	124	102	270	122
Al-Baha	89	407	106	97	322	115	–	–	–	94	339	113
Al-Jouf	101	413	130	–	–	–	104	356	130	103	362	130
Total	101	447	132	98	356	118	106	227	134	104	238	132

Sex ratio of 2010 is based on Preliminary Reports

In 2004, the sex ratio among non-native was also high at 227 males per 100 females and ranged from 216 to 384 in various governorates grouped as per the population size of native persons. Here too a widening sex ratio trend observed showing the increased requirements at domestic level, low skilled expatriate labor. That is, over the time, the tendency to employ low skilled and unskilled expatriates laborers increasing leading to demographic imbalances reflecting in the sex ratio. This might be an outcome of nationalization movement reducing skilled and professional expatriates.

It is important to note that sex ratio among the non-native persons declines with increasing number of native persons in a governorate. This may be due to the fact that small governorates bring expatriates in single—non family—status whereas larger governorates bring expatriates with family status; again depend upon the manpower requirements. Rapid urbanization (Telvizian 2009; Khraif 2007; United Nations 2006; Khraif 1994; Makki 1986) and influx of foreign labor (Collemore 2003; Khraif 2009; Wincker 1997; Alghamdi 1995) disturbs the sex ratio, depending upon the labor requirements; thus “Saudi Arabia and the nearby Qatar and Kuwait have the highest sex ratios in the world” (Parasuraman 2002). But the sex ratios are changing among the native Saudi community, as a result of environmental effects on reproductive health (Babay 2004), which is debatable. The increase in the overall sex ratio between 2004 and 2010 was mainly due to the larger increase in the sex ratio among non-native persons (11 percentage points) as compared to native person (3 percentage points). This widening sex ratio trend receives attention requiring caution at policy levels.

While the sex ratio changes among native persons is due to environmental effects; that of non-native persons is attributed to the labor importation (immigration) policies. Over all, the sex ratio was increased in all the regions between 2004 and 2010 with the highest increment in Al-Jouf region. This increase attributed to the trend followed by the non-native persons. But, sex ratio of non-native persons was declined in Al-Qaseem (26 points), Aseer (21 points) and Hail (12 points) regions between 2004 and 2010. Among the smaller governorates, Aseer region had experienced the highest decline in the sex ratio among the non-native persons between 2004 and 2010, followed by Al-Qaseem, Al-Baha, Makkah Al-Mokarramah and Al-Riyadh. However, in other regions of the small governorate group, we noticed an increase in the sex ratio among the non-native persons with maximum sex ratio change of 83 percentage points in Eastern Region and 78 percentage points in the Al-Jouf region. In the small governorates, we noticed a higher increase in the sex ratio among native persons in the Jazan region. Thus, the trend of sex ratio varies depending on the livelihood options and development activities regionally. The increasing infrastructure development during the intercensal period might have caused this trend.

In the medium governorate regions, a slight decline in the sex ratio among native persons was observed in three regions such as Al-Madina Al-Monawarah, Northern Borders, and Najran between 2004 and 2010 and the sex ratio was increased in other regions of the country during the same period. Among the non-native persons the sex ratio declined in the medium governorates in the regions of Aseer and Northen Borders. Highest increment in sex ratio among non-native persons in medium governorates was in Makkah Al-Mokarramah region, attributed to higher requirements of male labor force to serve the pilgrim population. In the large governorates, the difference in the sex ratio between 2004 and 2010 among the native persons was found to be small. Whereas, in case of non-native persons sex ratio was declined in the large governorates of Al-Qaseem and Aseer regions and it was increased in other regions. Increasing sex ratio cautions the policy making processes for its significance in reproductive health. On, the other hand, sex ratio of non-native persons remains high showing the gender specific labor oriented migration (Center for Population Studies 2012) to fulfill the labor requirements for efficient development programs. Thus, it is of a theoretical concern and a status issue, while including the labor immigrants in the sex ratio frame work.

### Ratio of population

An issue of concern to the fast developing Kingdom of Saudi Arabia is the exodus of expatriates hailing from Asia as well as Africa (Khraif 2009; Collemore 2003; Wincker 1997). There are 349 non-native per 1000 native population in the Kingdom (making 259 per 1000 persons), as of 2010 showing a decreasing trend compared to 2004 (372 out of Saudi and 271 out of total). Such a fast decreasing ratio indicates the changing labor markets and infrastructural development in the Kingdom (Table 4). In both 2004 and 2010, the ratio of non-native population to native population was small in medium governorates compared to small and large governorates. An overall ratio of 741:259 (native: non-native) observed in the Kingdom at governorate level, in 2010; the corresponding ratio during 2004 was 729:271 indicating a declining trend in the non-native population. The results indicate that population of non-natives decreased more in the medium and large governorates between 2004 and 2010. This gives hope for the efforts of nationalization movement in line with improving employment of national population.

**Table 4:** Ratio of population (per 1000) at governorate, across regions

Region	Non Saudi to Saudi	Saudi to total	Non Saudi to total	Non Saudi to Saudi	Saudi to total	Non Saudi to total	Non Saudi to Saudi	Saudi to total	Non Saudi to total	Non Saudi to Saudi	Saudi to total	Non Saudi to total
	<50,000			50,000–100,000			100,000+			Total		
*2004*												
Al-Riyadh	375	727	273	221	819	181	504	665	335	465	683	317
Makkah Al-Mokarramah	149	871	129	231	812	188	655	604	396	617	618	382
Al-Madina Al-Monawarah	113	898	102	120	893	107	377	726	274	322	756	244
Al-Qaseem	215	823	177	228	814	186	257	795	205	243	804	196
Eastern Region	265	790	210	–	–	–	318	759	241	315	761	239
Aseer	145	873	127	128	887	113	197	835	165	176	850	150
Tabouk	153	867	133	–	–	–	169	856	144	164	859	141
Hail	182	846	154	63	941	519	194	838	162	166	857	143
Northern Borders	157	864	136	157	864	136	175	851	149	167	857	143
Jazan	170	855	145	140	877	123	219	821	179	195	837	163
Najran	107	804	96	161	861	139	251	799	201	204	830	170
Al-Baha	126	888	112	161	861	139	–	–	–	151	869	131
Al-Jouf	158	864	136	–	–	–	176	850	150	174	852	148
Total	192	839	161	170	855	145	422	703	297	372	729	271
*2010*												
Al-Riyadh	361	735	265	208	828	172	464	683	317	444	693	307
Makkah Al-Mokarramah	147	872	128	261	793	207	586	630	370	557	642	358
Al-Madina Al-Monawarah	90	918	82	120	893	107	358	737	263	308	765	235
Al-Qaseem	196	836	164	–	–	–	251	799	201	237	808	192
Eastern Region	257	796	204	165	858	142	282	780	220	279	782	218
Aseer	198	834	165	117	895	105	181	847	153	167	857	143
Tabouk	156	865	135	118	894	106	160	862	138	156	865	135
Hail	195	837	163	–	–	–	194	838	162	169	856	144
Northern Borders	163	860	140	111	900	100	139	878	122	136	880	120
Jazan	47	956	44	159	863	137	219	820	180	188	842	158
Najran	111	900	100	150	870	130	262	792	208	213	825	175
Al-Baha	120	893	107	158	864	136	–	–	–	147	872	128
Al-Jouf	195	837	163	–	–	–	195	837	163	195	837	163
Total	199	833	166	152	868	132	338	720	280	349	741	259

The ratio of population in the regions for various governorates with different native population size shows lower non-native to native population was found in the governorates of Northern Borders followed by Al-Baha and Tabouk regions, whereas highest ratio was noticed in the regions of Makkah Al-Mokarramah and Al-Riyadh, in the year 2010 (Table 4). Governorates in regions with less urban population and urban infrastructure have lesser ratio; those urbanized regions have higher ratio: thus reflecting upon the demand for foreign laborers in those region’s developmental activities and life styles. Between 2004 and 2010, the non-native to native population ratio declined tremendously in Makkah Al-Mokarramah (60 points) followed by Eastern Region (36 points), Northern Borders (31 points) and Al-Riyadh (21 points). However, the ratio of non-native to native population was found to have increased during the period in governorates of few regions, such as Al-Jouf, Najran and Hail. This trend is crucial especially in the context of increasing number of expatriates in proportion with the native population. On the other hand, it shows the effect of nationalization strategy implementation.

In 2010, among the smaller governorates group, Al-Riyadh region followed by the Eastern Region had the highest ratio of non-native to native population and Jazan region had the lowest ratio. Between 2004 and 2010, in the small governorates, Jazan region had the largest decline in the non-native population followed by Al-Madina Al-Monawarah. But, governorates in Aseer, Al-Jouf and Hail region’s ratio of non-native to native population has increased during the period. In the medium governorates Makkah Al-Mokarramah and Al-Riyadh regions had the highest ratio of non-native population in 2010. We noticed an increase in the ratio of non-native to native population in two regions of medium governorates, namely Makkah Al-Mokarramah and Jazan between 2004 and 2010. In the larger governorates Makkah Al-Mokarramah and Al-Riyadh regions had the highest ratio of non-native to native population in 2010. There was an increase in the non-native population in larger governorates of Al-Jouf and Najran during the period 2004–2010.

### Households

In 2010, on an average there was 39,450 households per governorate in the Kingdom (4,655,127 in total); out of them 25,417 (2,999,218 in total) belonging to native person whereas 14,033 (1,655,909 in total) belonging to non-native person. The corresponding number of households in the year 2004 was 33,890 (3,999,011 in total), 23,466 (2,768,966) and 10,424 (1,230,045), respectively; suggesting an increasing trend over the period. This increase might be proportional to the population combined with the effect of nucleation of families. In all types of governorates, the native population had the highest number of households, which also agrees with the population size.

Apparently smaller governorates included lesser number of average households belonging to both native and non-native persons. Overall, in 2004 the average size ranged between 2361 and 9288 households in the smaller governorates (1870–7487 in case of native persons and 491–2157 in case of non-native persons). On the other hand, medium sized governorates included 13,123 households, on an average and ranged between 9318 and 18,707 (7804–12,015 in case of native persons and 1299–4231 in case of non-native persons). The average number of households in the larger governorates was 103,360 and it ranged between 21,666 and 271,628.

In total, the mean number of households per governorates varied from 10,679 (Najran) to 110,743 (Makkah Al-Mokarramah), as of 2010 and the mean for 2004 was 8742 (Najran) and 98,159 (Makkah Al-Mokarramah). The results also suggests that governorates in Al-Riyadh and Eastern Region had comparatively higher number of households and governorates whereas Al-Baha, Northern Borders, Jazan and Al-Qaseem had comparatively lesser number of households (Table 5). Among the small governorates Aseer and Al-Jouf regions had the highest average number of households in 2010. However, in 2004 Al-Madina Al-Monawarah and Aseer regions found to have the highest average number of households. In 2010, minimum number of average households in the small governorates was identified in Jazan and Najran, and in 2004 the same was observed in Najran and Al-Riyadh regions. Similarly, in 2010 Makkah Al-Mokarramah, Hail and Al-Riyadh regions had the high mean number of households among the medium size governorates, whereas Eastern Region, Jazan and Tabouk regions had governorates with low mean number of households. However, in 2004 Al-Qaseem, Makkah Al-Mokarramah and Al-Riyadh regions had the maximum mean number of households and Jazan and Northern Borders regions are found to have the minimum average number of households in a governorate. In both 2010 and 2004, larger governorates in the regions of Makkah Al-Mokarramah and Al-Riyadh had highest mean number of households. In 2010, lowest mean number of households was found in the governorates of Northern Borders and Jazan regions and in 2004 it was minimum in the governorates of Al-Jouf and Northern Borders regions.

**Table 5:** Average number of households in governorate across regions

Region	Saudi	Non Saudi	Total	Saudi	Non Saudi	Total	Saudi	Non Saudi	Total	Saudi	Non Saudi	Total
	<50,000			50,000–100,000			100,000+			Total		
*2004*												
Al-Riyadh	3235	1984	5219	10,632	4231	14,864	175,544	96,084	271,628	31,301	16,773	48,074
Makkah Al-Mokarramah	5747	1778	7525	11,063	3836	14,899	134,539	87,558	222,097	60,296	37,863	98,159
Al-Madina Al-Monawarah	7487	1802	9288	9472	1299	10,771	80,657	31,030	111,686	28,676	10,081	38,757
Al-Qaseem	3986	1472	5458	14,348	4359	18,707	38,461	14,114	52,575	11,196	4033	15,229
Eastern Region	4750	1621	6371	–	–	–	62,370	21,286	83,656	36,179	12,348	48,527
Aseer	6626	1609	8235	10,532	2473	13,005	38,425	11,219	49,643	18,853	5172	24,025
Tabouk	5880	1311	7191	–	–	–	67,500	15,024	82,524	16,150	3596	19,746
Hail	4236	2157	6393	12,015	1978	13,993	39,673	11,833	51,506	15,040	4531	19,571
Northern Borders	4656	1091	5747	7915	1811	9726	18,073	4313	22,386	10,215	2405	12,620
Jazan	5213	1282	6494	7804	1514	9318	21,251	5948	27,199	10,165	2648	12,813
Najran	1870	491	2361	9620	1574	11,194	31,360	13,211	44,571	6525	2216	8742
Al-Baha	4620	1332	5952	9391	2587	11,978	–	–	–	7346	2049	9396
Al-Jouf	4312	1361	5673	–	–	–	16,672	4994	21,666	12,552	3783	16,335
Total	4571	1502	6073	10,213	2909	13,123	70,013	33,346	103,360	23,466	10,424	33,890
*2010*												
Al-Riyadh	3659	2772	6432	10,034	4836	14,870	120,240	84,889	205,129	34,080	23,714	57,794
Makkah Al-Mokarramah	6313	2063	8375	12,629	5831	18,460	143,066	106,957	250,023	64,346	46,397	110,743
Al-Madina Al-Monawarah	6552	1432	7984	9477	2568	12,045	83,756	42,809	126,565	30,282	13,903	44,185
Al-Qaseem	4658	1804	6463	–	–	–	32,701	17,624	50,324	12,306	6119	18,425
Eastern Region	4624	2071	6695	7417	3035	10,452	66,823	30,198	97,021	38,805	17,501	56,305
Aseer	7185	3214	10,399	9706	2653	12,359	37,092	13,257	50,349	20,907	7118	28,025
Tabouk	5313	1876	7189	8756	2101	10,857	73,465	20,140	93,605	17,245	4957	22,203
Hail	4758	2895	7653	14,609	2097	16,706	44,023	18,620	62,643	17,037	6627	23,664
Northern Borders	4850	1407	6257	8965	2495	11,460	19,020	6170	25,190	10,945	3357	14,302
Jazan	2298	235	2533	8392	2211	10,603	21,541	8150	29,690	10,843	3484	14,327
Najran	1982	576	2558	10,779	2350	13,129	36,388	20,566	56,954	7382	3297	10,679
Al-Baha	5384	1796	7180	9875	3612	13,487	–	–	–	7950	2834	10,784
Al-Jouf	5868	2634	8502	–	–	–	21,785	9176	30,962	16,480	6996	23,475
Total	4355	1901	6257	9697	3100	12,797	68,644	40,601	109,245	25,417	14,033	39,450

Number of households belonging to both native and non-native persons per governorate had increased in all the regions during the period 2004–2010. We noticed a negligible decline in case of households of native persons in smaller governorates (Eastern Region and Tabouk) and in medium sized governorates (Al-Riyadh and Aseer). However, a noticeable decline was observed in smaller governorates of Jazan and larger governorates of Al-Riyadh, Al-Qaseem and Aseer.

### Number of persons per household

Number of households depends upon the number of persons in a governorate. Number of persons per household was 5.60 in the Kingdom, as of 2010; 6.45 in case of native persons and 4.08 in case of non-native persons, as compared to 5.48, 6.08 and 4.12, respectively, in 2004 (Table 6). The result suggests that the average household size was increased mainly among the native population, despite the effects of urbanization and modernization. No difference was observed across the various types of governorates on the number of persons per household, except in case of non-native persons. Larger governorates found to have bigger size households among non-native persons, an indication of family status and housing issues, attached to labor VISA (provisions of family status and accommodation facilities). The situation improving with relaxed rules and regulations.

**Table 6:** Average number of persons per households in governorate across regions

Region	Saudi	Non Saudi	Total	Saudi	Non Saudi	Total	Saudi	Non Saudi	Total	Saudi	Non Saudi	Total
	<50,000			50,000–100,000			100,000+			Total		
*2004*												
Al-Riyadh	6.19	3.79	5.28	6.46	3.58	5.64	5.87	5.41	5.71	5.95	5.17	5.68
Makkah Al-Mokarramah	5.87	2.82	5.15	5.31	3.53	4.85	4.9	4.93	4.92	4.95	4.87	4.92
Al-Madina Al-Monawarah	6.28	2.95	5.63	5.48	4.78	5.39	5.61	5.49	5.58	5.7	5.22	5.58
Al-Qaseem	6.61	3.85	5.87	6.58	4.94	6.19	6.66	4.67	6.12	6.64	4.48	6.06
Eastern Region	7.1	5.52	6.7	–	–	–	6.38	5.95	6.27	6.42	5.92	6.29
Aseer	6.65	3.97	6.13	6.3	3.43	5.75	6.32	4.27	5.85	6.34	4.08	5.85
Tabouk	6	4.12	5.66	–	–	–	6.19	4.69	5.92	6.13	4.52	5.84
Hail	7.54	2.7	5.91	7.4	2.82	6.76	7.53	4.89	6.93	7.51	4.15	6.73
Northern Borders	7.75	5.2	7.27	8.01	5.5	7.54	7.76	5.68	7.36	7.83	5.56	7.4
Jazan	7.15	4.93	6.72	7.47	5.39	7.13	6.81	5.32	6.49	6.98	5.22	6.62
Najran	6.59	2.68	5.78	6.53	6.45	6.52	6.77	4.03	5.96	6.69	4.02	6.01
Al-Baha	6.62	2.55	5.77	8.21	4.47	5.79	–	–	–	6.38	3.46	5.75
Al-Jouf	8.03	4.01	7.06	–	–	–	8.2	4.83	7.42	8.18	4.73	7.38
Total	6.58	3.84	5.9	6.43	3.84	5.85	5.84	5.18	5.63	5.97	5	5.67
*2010*												
Al-Riyadh	6.16	2.94	4.77	6.6	2.85	5.38	6.64	4.37	5.7	6.61	4.22	5.63
Makkah Al-Mokarramah	5.81	2.62	5.03	5.39	3.05	4.65	5.53	4.34	5.05	5.54	4.28	5.01
Al-Madina Al-Monawarah	6.73	3.76	5.02	5.86	2.59	5.16	6.15	4.3	5.52	6.11	4.1	5.48
Al-Qaseem	6.6	3.34	5.69	–	–	–	7.25	3.38	5.9	7.07	3.37	5.84
Eastern Region	7.5	4.3	6.51	7.76	3.13	6.41	6.92	4.32	6.11	6.96	4.3	6.14
Aseer	6.25	2.77	5.17	6.62	2.84	5.81	6.45	3.27	5.61	6.48	3.17	5.64
Tabouk	6.49	2.87	5.55	6.26	3.08	5.65	6.53	3.82	5.95	6.5	3.53	5.84
Hail	7.39	2.37	5.49	6.66	2.9	6.19	7.72	3.54	6.48	7.45	3.23	6.27
Northern Borders	8.48	4.78	7.65	7.86	3.14	6.83	8.55	3.67	7.36	8.35	3.7	7.26
Jazan	6.87	3.12	6.52	7.6	4.58	6.97	7.29	4.22	6.44	7.39	4.32	6.64
Najran	6.69	2.56	5.76	6.81	4.69	6.43	7.05	3.27	5.68	6.93	3.3	5.81
Al-Baha	6.26	2.24	5.25	6.42	2.77	5.44	–	–	–	6.37	2.63	5.39
Al-Jouf	6.93	3.01	5.71	–	–	–	7.29	3.37	6.13	7.25	3.33	6.09
Total	6.58	3.01	5.47	6.65	3.17	5.8	6.41	4.21	5.6	6.45	4.08	5.6

Number of persons per household in a governorate decreased during 2004–2010, especially in Makkah Al-Mokarramah, Al-Riyadh and Eastern Region, lowest increase was observed in Al-Baha, Jazan and Northern Borders.

### Coefficient of variation

An attempt is made to extract variations in a few population dimensions of governorates grouped into three based on the number of native persons as shown in the Table 1. Indications of higher levels of variability within the governorates were observed. Variations decreased from 2004 to 2010 in case of all indicators for both small and medium sized governorates but the reverse is true in case of large governorates. Change in male (−9.7 points) and female population of native (6.5 points) and thus the total native population (−4.4 points) indicates the differential growth of population at local geographic units that reflecting migrations more than other factors like natural increase (Table 7). On the other hand, the decrease in the coefficient of variation indicates a movement towards balance. It may also be due to redefinitions and reclassification of governorates, during the intercensal period. The overall scenario is a mix of both increases and decreases. An increase of coefficient of variation noted in case of non-native population (12.5 points), non-native households (8.2 points), male non-native population (4.9 points), male total population (0.6 points) and female total population (1.3 points) indicating an unplanned and unexpected change in population, especially of non-native (foreigners).

**Table 7:** Coefficient of variation (CV) for various population dimensions across groups of governorates (%)

	<50,000			50,000–100,000			100,000+			Total		
	2004	2010	Difference	2004	2010	Difference	2004	2010	Difference	2004	2010	Difference
Saudi population	228.2	44.8	−183.4	147.0	20.0	−126.9	102.1	136.7	34.7	234.9	230.4	−4.4
Non Saudi population	318.2	69.0	−249.2	175.0	45.8	−129.2	108.9	214.6	105.7	362.2	374.7	12.5
Total population	251.4	45.4	−206.0	151.5	20.6	−130.9	101.8	156.3	54.5	265.0	264.1	−1.0
Saudi households	239.3	46.1	−193.2	141.0	22.1	−118.9	105.8	140.3	34.5	249.0	236.3	−12.6
Non Saudi households	305.8	63.2	−242.6	152.5	52.6	−99.9	97.9	200.6	102.7	333.5	341.7	8.2
Total households	258.1	46.3	−211.8	143.7	25.0	−118.7	103.3	161.3	58.1	272.0	271.4	−0.6
Male Saudi population	232.8	43.5	−189.3	151.3	20.2	−131.2	102.9	136.7	33.8	240.9	231.2	−9.7
Female Saudi population	223.6	46.4	−177.2	142.8	20.3	−122.5	101.3	131.9	30.6	228.8	222.3	−6.5
Male non Saudi population	307.6	73.0	−234.6	172.5	54.1	−118.5	107.4	201.9	94.4	344.4	349.3	4.9
Female non Saudi population	344.9	76.8	−268.0	184.7	56.3	−128.3	115.0	226.5	111.4	409.7	405.0	−4.7
Male total population	257.3	46.9	−210.4	156.4	23.1	−133.3	102.4	161.3	58.9	272.9	273.4	0.6
Female total population	244.1	46.7	−197.4	145.9	20.5	−125.4	101.7	151.7	50.1	255.4	256.7	1.3
Ratios (%)—median centered												
Population (Saudi/non Saudi)	58.0	142.7	84.7	75.0	40.0	−35.0	54.1	48.8	−5.3	70.0	101.8	31.8
Population (Saudi/total)	11.6	9.7	−1.9	6.0	5.4	−0.6	9.3	11.3	2.0	10.0	9.5	−0.5
Households (Saudi/non Saudi)	54.2	176.0	121.8	58.0	43.4	−14.6	43.1	56.6	13.5	54.1	115.9	61.8
Households (Saudi/total)	13.0	15.8	2.8	8.8	10.0	1.2	12.8	12.4	−0.4	12.1	13.7	1.6
Saudi population (male/female)	8.0	49.8	41.8	6.7	6.5	−0.2	7.4	6.7	−0.7	7.6	32.8	25.2
Non Saudi population (male/female)	38.0	40.3	2.3	18.2	36.1	17.9	25.7	39.7	14.0	31.7	42.3	10.6
Total population (male/female)	17.3	39.0	21.7	10.1	15.0	4.9	16.3	21.1	4.8	16.2	30.1	13.9

The male–female distribution for the year 2010 taken from the Preliminary Reports, while the total from Final Report

Small governorates have coefficients of variation increasing for all indicators except native population and female non-native population, indicating an increasing variability across governorates within the category: non-native receiving greater attention for their increasing variability in terms of population, especially male. This reflects an imbalance of population size in the governorates and which is increasing. Imbalances remain larger in case of population and households of non-native. In the medium sized governorates, coefficient of variation decreased in 2010, except in case of non-naitve population (both male and female), indicating a movement towards balance. In the large governorates, the coefficient of variation was increased for all the indicators. The large governorates having urban agglomerations and increasing levels of infrastructure development pull people for occupational purposes leading to an increase in the residential population.

Median centered coefficient of variation of population shows good performance in the ratio of population native to total and of households. The balance of naitve male to female during 2004 (7.6) has lost during 2010 (32.8), reflecting a rapid change in population settlements probably due to rural to urban migration of males. A rapid imbalance observed in case of native to non-native population, indicating immigration trends and urbanization. A growing imbalance of sex is also noted.

## Conclusions and implications

Population size of an administrative unit influences local level demographics—sex ratio, ratio of native to non-native population, number of households and number of persons per household. Smaller administrative areas have a demographic profile different from that of larger areas, as seen from the data of Saudi Arabia. Population pressure increases developmental and infrastructural pressures, in turn, influencing the population characteristics. Larger governorates have more stable and developed administrative systems and structures, thus, having modern infrastructure and characteristics showing a balanced demography, in terms of sex ratio and persons per household. On the contrary, the medium sized units—the developing units—have accelerated growth of infrastructure requiring additional manpower, which is fulfilled through bringing people either from rural areas or from outside the country, thus altering population characteristics in line with masculinity, declining ratio of native to non-native population, changing household composition and thus persons per household. At the same time, small units usually are traditional, in terms of livelihoods and lifestyles that influences demographic structure and characteristics, but with exemption of qualifying as independent units.

The situation in Saudi Arabia follows a similar trend, but with the exceptions that the Kingdom has a huge share of foreign labor force; a majority of them lives in large administrative units. The exodus of foreign labor shows a diversion of demographic trends; high sex ratios, lesser proportion of native and smaller households. However, native population has characteristics close to the expectations—sex ratio close to 100 and has more than 6 persons per household.

Wide variations of administrative units in population size (male–female; native-non-native) and number of households, show an inequitable allocation caused due to the criterion set for qualifying an area to be recognized as an administrative unit. Reclassifications, in future, to equalizing units adopting a strict criterion (population size, geographic area combined with infrastructure development) shall balance this issue.

Changes happened from 2004 to 2010, on demographic characteristics of administrative areas reduce the gap, with exceptions in case of non-native population who constitutes the male dominated skewed adult labor force. The unprecedented increase in their numbers, despite the regulations in labor and immigration laws, during 2004–2010, reduces the ratio of native to total population, especially in Makkah Al-Mokarramah, Al-Riyadh, the Eastern Region and Al-Madina Al-Monawarah regions.

This research made possible through analyzing national census carried out during 2004 and 2010 explored few basic demographics with the aim of exposing the Kingdom’s grass root level planning and development as reflected in the local level demographic characteristics. Findings of this research have implications on both local level development plans as well as on improving or manipulating national demographic homogeneity. Developmental efforts are at a peak in the Kingdom even though with varying intensities across regions. While the East West Corridor (from Alkhobar to Jeddah governorate) develops faster, areas on the southern and northern side develop slowly, as shown by the data set, which may be addressed through population redistribution efforts with equitable developmental efforts. For the purpose, it is vital to improve the livelihoods and public infrastructure at poorly performing governorates, with an aim to integrate them into the main stream of the Kingdom so that people move to such localities for employment and residential purposes. Such efforts shall pave way for manipulating demographic characteristics like sex ratio, native-non-native ratio and persons per household—the three basic demographics discussed in this paper, which will raise the image of the Kingdom among the developed demographies of the world. It is also of importance to continue the collection of grass root level demographic data through Census and sample surveys at periodic intervals to create population based grass root level developmental plans, which will change the macro level indicators.
